# The role of Rak in the regulation of stability and function of BRCA1

**DOI:** 10.18632/oncotarget.5717

**Published:** 2015-10-14

**Authors:** Jung-Lye Kim, Geun-Hyoung Ha, Loredana Campo, Mitchell F. Denning, Tarun B. Patel, Clodia Osipo, Shiaw-Yih Lin, Eun-Kyoung Breuer

**Affiliations:** ^1^ Oncology Institute, Cardinal Bernardin Cancer Center, Stritch School of Medicine, Loyola University Chicago, Maywood, IL 60153, USA; ^2^ Department of Radiation Oncology, Stritch School of Medicine, Loyola University Chicago, Maywood, IL 60153, USA; ^3^ Department of Pathology, Stritch School of Medicine, Loyola University Chicago, Maywood, IL 60153, USA; ^4^ Department of Molecular Pharmacology and Therapeutics, Stritch School of Medicine, Loyola University Chicago, Maywood, IL 60153, USA; ^5^ Department of Systems Biology, University of Texas MD Anderson Cancer Center, Houston, TX 77030, USA

**Keywords:** BRCA1, Rak, tyrosine phosphorylation, DNA damage response, genomic stability

## Abstract

BRCA1 is an important player in the DNA damage response signaling, and its deficiency results in genomic instability. A complete loss or significantly reduced BRCA1 protein expression is often found in sporadic breast cancer cases despite the absence of genetic or epigenetic aberrations, suggesting the existence of other regulatory mechanisms controlling BRCA1 protein expression. Herein, we demonstrate that Fyn-related kinase (Frk)/Rak plays an important role in maintaining genomic stability, possibly in part through positively regulating BRCA1 protein stability and function via tyrosine phosphorylation on BRCA1 Tyr1552. In addition, Rak deficiency confers cellular sensitivity to DNA damaging agents and poly(ADP-ribose) polymerase (PARP) inhibitors. Overall, our findings highlight a critical role of Rak in the maintenance of genomic stability, at least in part, through protecting BRCA1 and provide novel treatment strategies for patients with breast tumors lacking Rak.

## INTRODUCTION

The tumor suppressor BRCA1 plays essential roles in various cellular processes, including cell cycle checkpoint control [1], DNA repair [2], apoptosis [3, 4], transcriptional regulation [5, 6] and chromatin remodeling [7]. Hereditary harmful *BRCA1* mutations have been linked with an increased risk of developing breast cancer [8, 9]. Interestingly, studies have shown that about 30∼40% of patients with sporadic breast cancer have complete loss or significantly reduced expression of BRCA1 protein despite carrying an intact *BRCA1* gene [10–15]. Loss of BRCA1 expression and/or function has been shown to be significantly associated with highly aggressive metastatic breast tumor phenotype [16, 17] and a poor prognosis [18]. Growing evidence suggests that BRCA1 expression is regulated at multiple levels by transcription factors, microRNA (miRNA) and posttranslational modifications [12, 19–22]. Particularly, disruption of BRCA1 protein stability represents a very attractive mechanism to be studied, however, the molecular mechanisms responsible for the stability of BRCA1 protein remain to be elucidated.

Rak belongs to the Src tyrosine kinase family [23], however, unlike Src, mounting evidence suggests that Rak functions as a tumor suppressor in human cancer [24–26]. For instance, *Rak* is located on chromosome 6q21–23, a region showing loss of heterozygosity (LOH) in 30% of breast cancer [27, 28] and frequent deletion in non-small cell lung cancers (NSCLCs) [29, 30] and chronic lymphocytic leukemia (CLL) [31]. *Iyk*, the mouse homologue of Rak [32], is lost during breast carcinogenesis [33]. Rak has inhibitory effects on cell proliferation [24–26, 34] and epidermal growth factor receptor (EGFR) signaling in a variety of cancer cells [35]. Rak also induces apoptosis and G1 arrest possibly through decreasing hyper-phosphorylated retinoblastoma (pRB) [34], a known interacting partner of Rak [26], and E2F1, and reduces migratory and invasive capabilities of glioma cells by suppressing the c-Jun N-terminal kinase (JNK)/c-Jun signaling pathway [36] and by enhancing the formation of the N-cadherin-β-catenin complex [37]. We have also previously demonstrated that Rak deficiency induces tumorigenic potential of non-tumorigenic immortalized human mammary epithelial cells both *in vitro* and *in vivo*, and that Rak-mediated tyrosine phosphorylation of the tumor suppressor phosphatase and tensin homolog (PTEN) is critical for its protein stability and function [25]. Although we and others have shown a critical role for Rak as a tumor suppressor, we still do not know the full extent and significance of Rak in breast cancer.

In this study, we demonstrate a novel mechanism by which Rak contributes to the maintenance of genome stability. Importantly, Rak-mediated phosphorylation of BRCA1 on Tyr1552 is critical for the stability and function of BRCA1. Moreover, Rak deficiency confers increased cellular sensitivity to DNA damaging agents, including ionizing radiation (IR) and Cisplatin as well as poly(ADP-ribose) polymerase (PARP) inhibitor. Together, our study suggests Rak as a new guardian of genome stability and a potential prognostic indicator that predicts therapeutic responses to radiotherapy, chemotherapy and PARP inhibitor therapy.

## RESULTS

### Rak deficiency causes spontaneous DNA damage due to defective DSB repair

**Figure 1:**
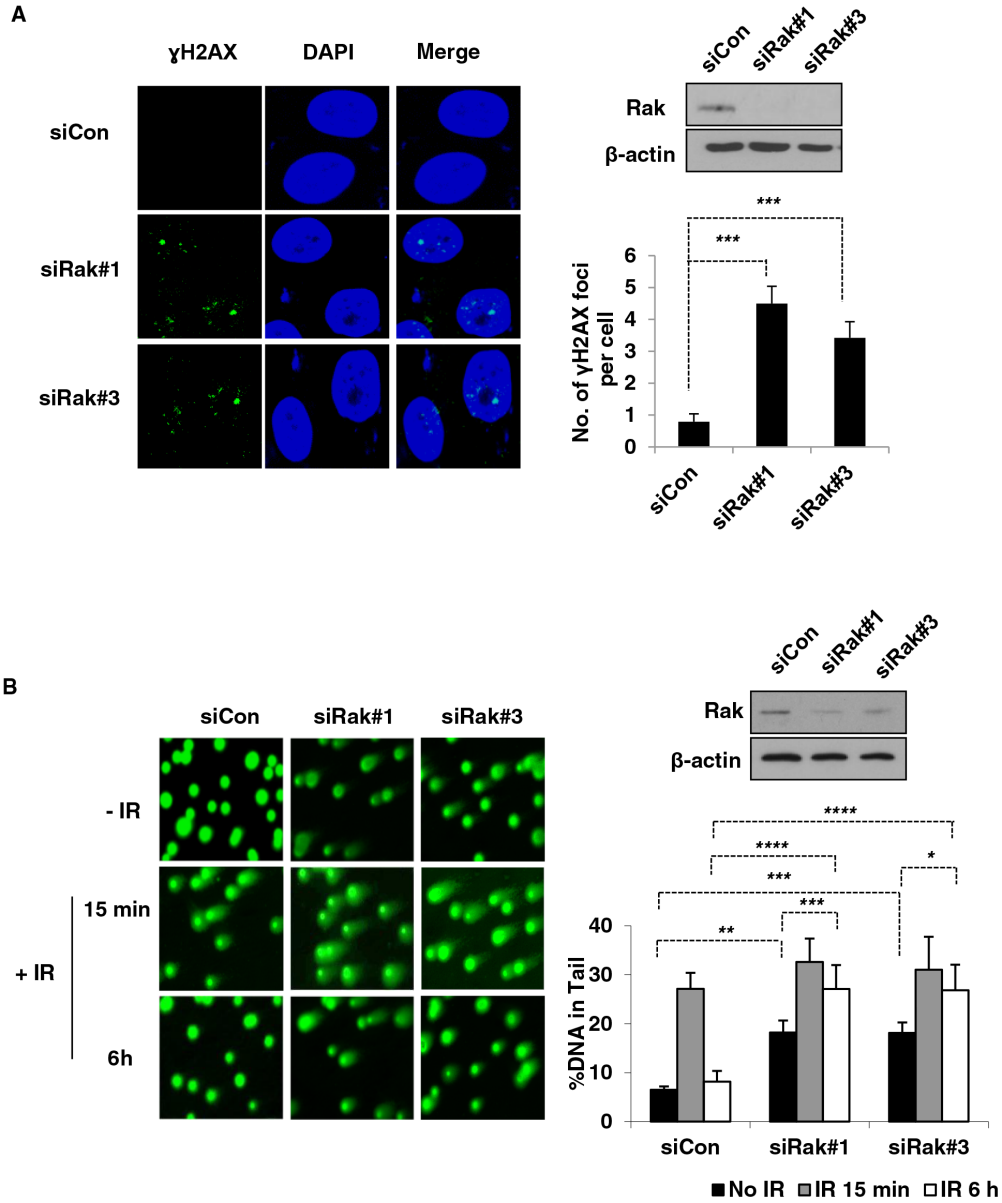
Rak deficiency leads to the accumulation of DSBs due to a defect in DSB repair MCF10A cells were transfected with either control siRNA or Rak siRNAs and 48 h after transfection, cells were subjected to immunofluorescence staining and comet assays. **A.** Foci formation of γH2AX was assessed by immunofluorescence staining. Cells were stained with an anti-phospho-histone H2AX^Ser139^ antibody, followed by incubation with Alexa 488 Fluor-conjugated secondary antibody. DAPI staining was carried out for visualizing nuclei. **B.** Incidence of DSBs and repair efficiency were measured by the neutral pH comet assay. At 15-min and 4-hour post-IR, cells were subjected to the neutral pH comet assay. The percent of tail DNA from at least 200 cells was scored. Result represents the mean ± SD of at least three independent experiments. *p < 0.05; **p < 0.001; ***p < 0.005; ****p < 0.0005

Based upon our previous findings that Rak positively regulates the stability and function of PTEN [25], together with mounting evidence demonstrating a role of PTEN in the maintenance of genomic stability [38, 39], we hypothesized that Rak, too, may be involved in maintaining genomic stability. To test our hypothesis, we first performed the γH2AX focus formation assay to examine the induction of DNA double-strand breaks (DSBs) in the absence of Rak. As shown in Figure 1A, Rak-depleted MCF10A cells (siRak#1 and #3) exhibited an increase in the number of γH2AX foci compared to control cells, suggesting that Rak deficiency causes an accumulation of spontaneous DNA DSBs. The induction of DSBs in the absence of Rak was also confirmed by the neutral comet assay. The fraction of DNA in comet tails (% DNA in Tail, indicative of relative amount of DNA damage) of Rak-depleted MCF10A cells (siRak#1 and #3) was significantly higher than control cells in the absence of IR (Figure 1B, top row). At 15 min post-IR, all cells exhibited an increase in DNA damage, indicating IR-induced DSBs (Figure 1B, middle row). At 6 h post-IR, control cells had almost completed DNA repair process, whereas Rak-depleted MCF10A cells retained DSBs (Figure 1B, bottom row). These data suggest that Rak deficiency causes an accumulation of spontaneous DNA damage and a defect in the repair of DSBs.

### Rak deficiency results in impaired DSB repair and checkpoint control

**Figure 2:**
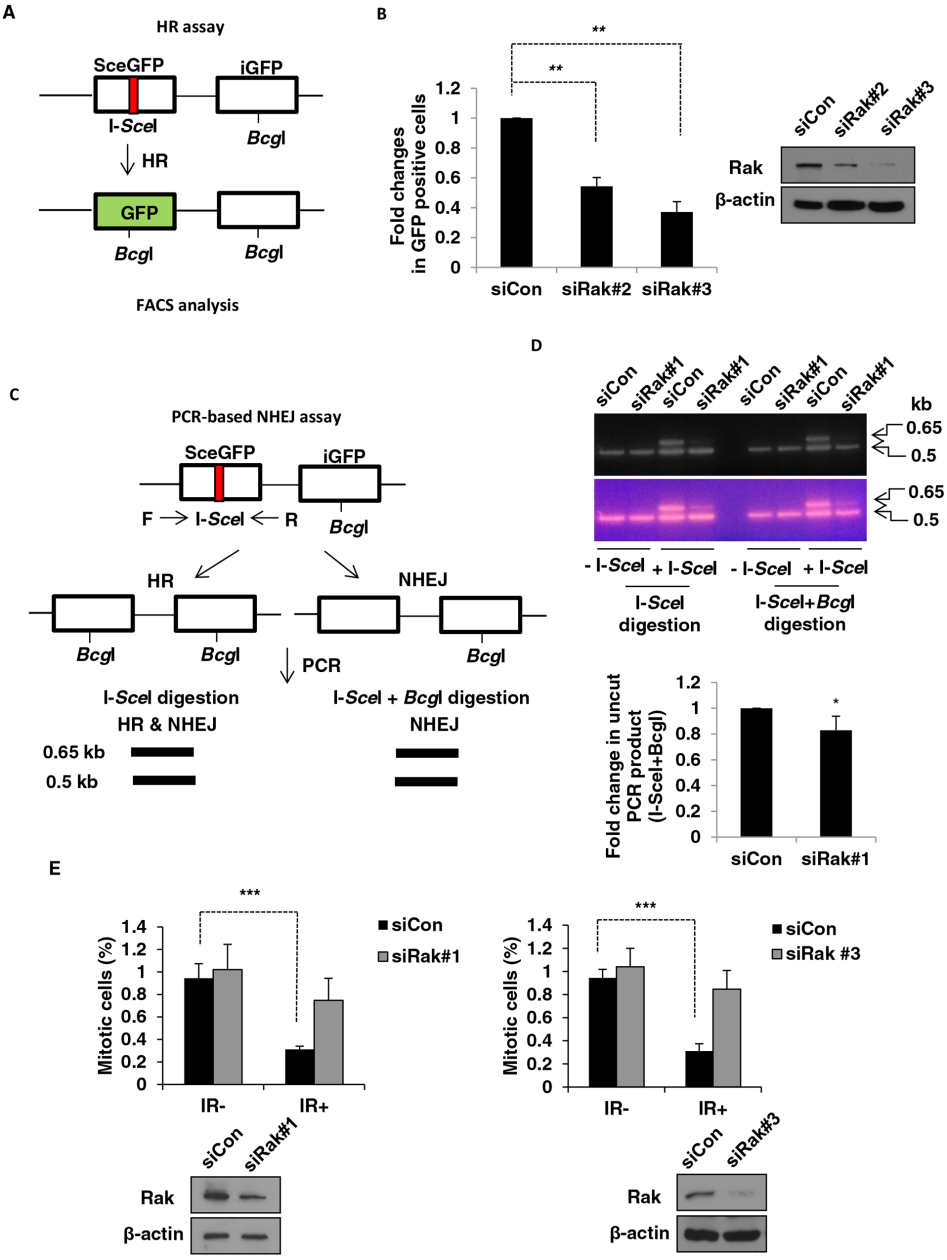
Rak deficiency results in impaired DSB repair and G2/M checkpoint **A.** A schematic diagram depicts the HR assay. MCF10A cells were transfected with either control siRNA or Rak siRNAs. **B.** Twenty four hours after transfection, pDR-GFP along with either pCBASce (I-*Sce*I expressing vector) or pCACGS (empty vector) were co-transfected. Cells were harvested to analyze HR-repaired GFP-positive cells by FACS. **C.** A schematic diagram depicts the PCR-based NHEJ assay. **D.** Twenty four hours after transfection, pDR-GFP along with either pCBASce or pCACGS were co-transfected. The genomic DNA was extracted for PCR amplification, digested with I-*Sce*I (HR and NHEJ) or I-*Sce*I+*Bcg*I (NHEJ) and subjected to agarose gel electrophoresis. The intensity of bands was quantified using NIH imageJ software (http://imagej.nih.gov/ij/). **E.** Cells were irradiated with 3 Gy of IR and then incubated for 1 h. Mitotic cells were determined by staining with an anti-phospho-histone H3 antibody followed by incubation with FITC-conjugated secondary antibody and propidium iodide. The percentage of cells in M-phase was analyzed by FACS. Result represents the mean ± SD of at least three independent experiments. *p < 0.05; **p < 0.001; ***p < 0.0005

In mammalian cells, there are two major DSB repair pathways, homologous recombination (HR) and non-homologous end joining (NHEJ) under the control of the checkpoint mechanism [40–42]. To further investigate the role of Rak in DSB repair, we first examined HR efficiency by employing the pDR-GFP reporter system (Figure 2A) [43–45] with modifications [46–48]. As shown in Figure 2B, Rak deficiency resulted in a significant decrease (50∼60%) in GFP-positive cells repaired by HR. A similar result was also observed in Rak-depleted U2OS cells (Supplementary Information, Figure S1A). On the other hand, ectopic expression of Rak promoted HR-mediated DSB repair (Supplementary Information, Figure S1B). To determine the involvement of Rak in NHEJ-mediated DSB repair, the PCR-based assay (Figure 2C) [44, 45, 49, 50] was carried out. As shown in Figure 2D, NHEJ repair efficiency was also slightly decreased (15∼20%) in the absence of Rak. These data suggest that Rak is involved in both the HR and NHEJ-mediated DSB repair.

The comparison of phosphorylation of histone H3 at Ser^10^ after exposure to IR revealed that the majority of Rak-depleted MCF10A cells underwent mitosis while the mitotic population of control cells was significantly decreased (Figure 2E), suggesting a defect in G2/M checkpoint. A similar result was also observed in Rak-depleted HEK293T cells (Supplementary Information, Figure S2). Together, our data reveal a critical role for Rak in DSB repair and G2/M cell cycle checkpoint.

### Rak associates with and positively regulates BRCA1 protein stability

**Figure 3:**
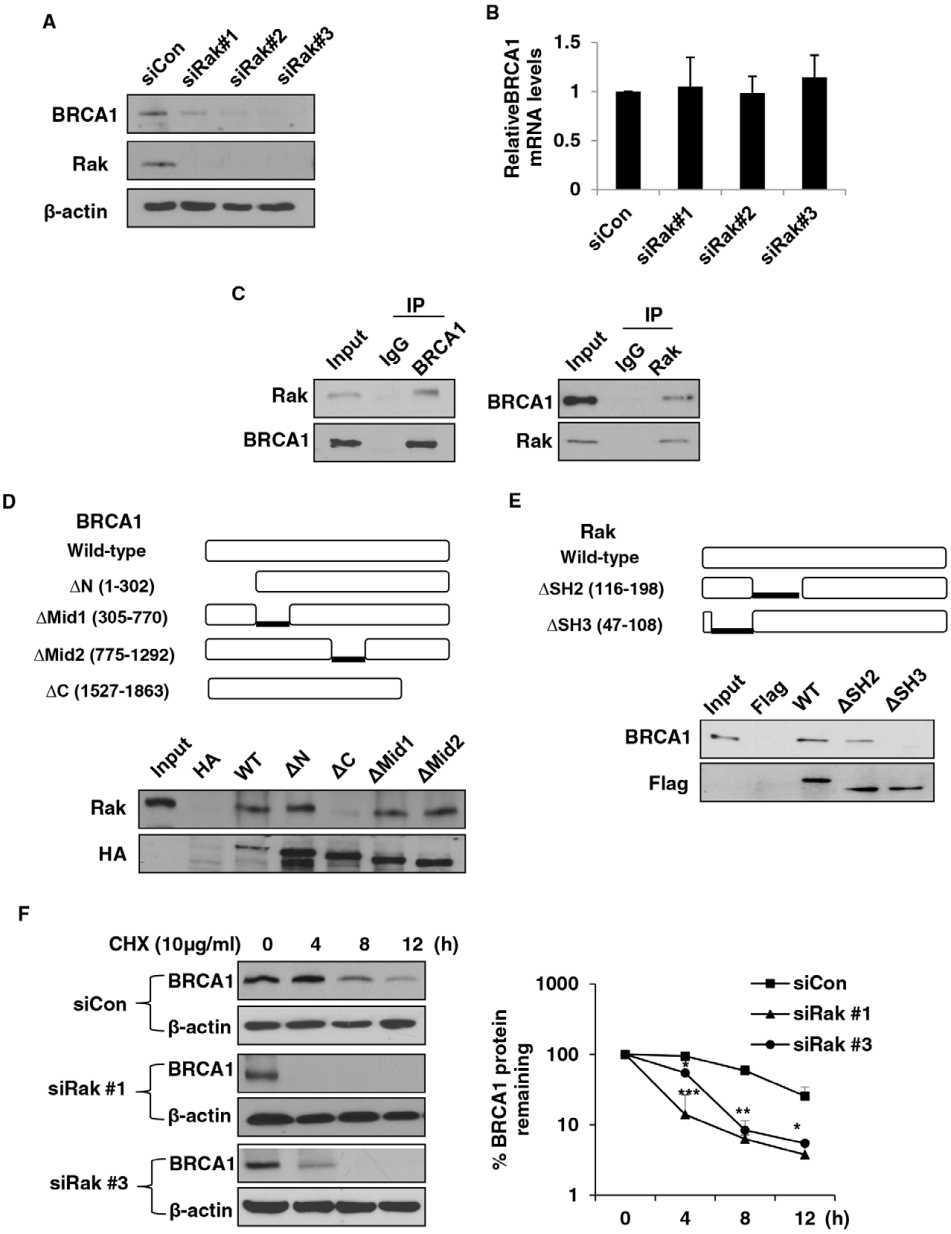

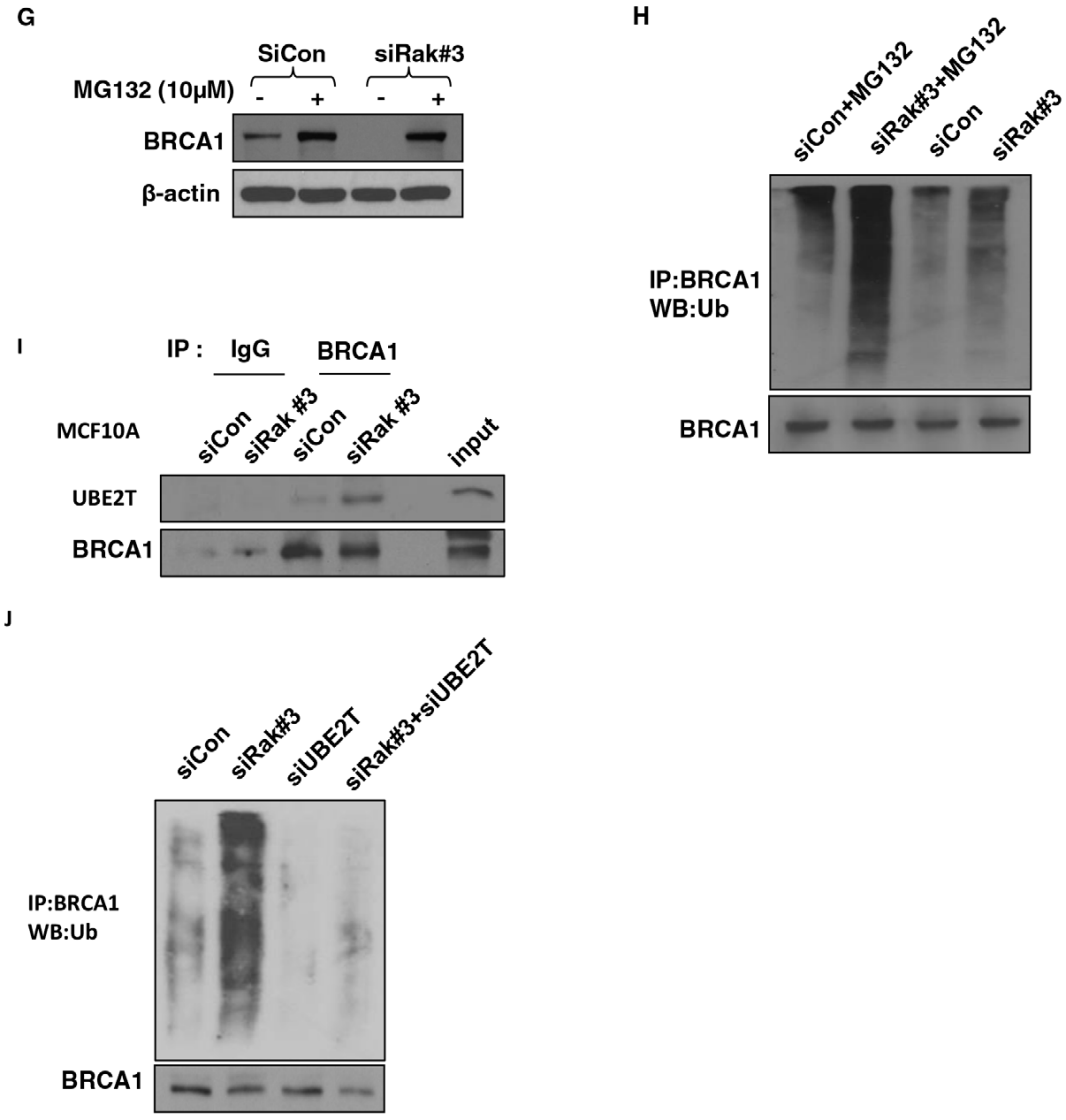

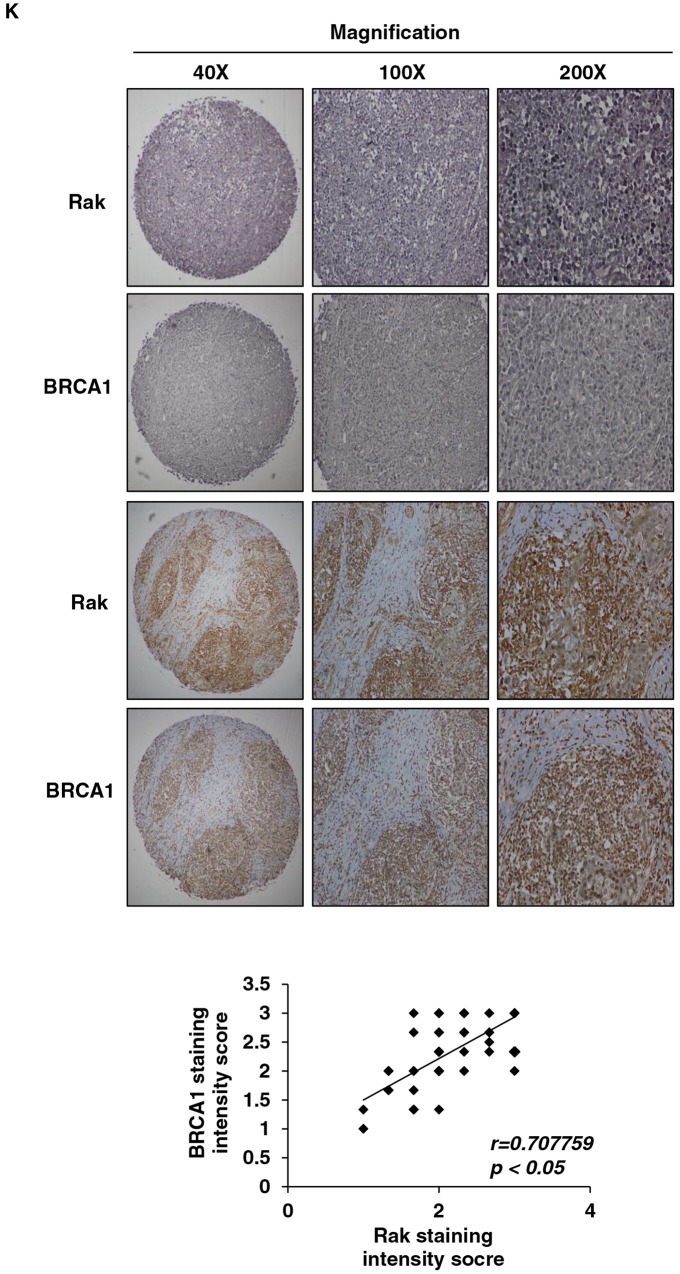
Rak interacts with BRCA1 and positively regulates BRCA1 protein stability MCF10A cells were transfected with either control siRNA or Rak siRNAs for 48 h. Cells were harvested, lysed and subjected to **A.** western blot and **B.** quantitative real-time PCR analysis. **C.** Rak interacts with BRCA1. Lysates from MCF10A cells were immunoprecipitated with an anti-Rak or anti-BRCA1 antibody and immunoblotted with an anti-BRCA1 or anti-Rak antibody, respectively. **D.** Mapping the BRCA1 binding domain of Rak. Plasmid constructs for wild-type (WT) and deletion mutants (Δ1–302 [ΔRING], Δ305–770 [ΔN-terminal NLS], Δ775–1292 [ΔC-terminal NLS] and Δ1527–1863 [ΔBRCT]) of HA-BRCA1 [64] were transfected into 293T cells, pulled down with an anti-HA antibody and subjected to western blot analysis with an anti-Rak antibody. **E.** Mapping the Rak binding domain of BRCA1. Plasmid constructs for WT and deletion mutants (ΔSH2 and ΔSH3) of Flag-Rak were transfected into 293T cells, pulled down with anti-Flag M2 beads and subjected to western blot analysis with an anti-BRCA1 antibody. **F.** Cells were incubated with 10 μg/ml of cycloheximide (CHX) for the indicated periods of time to inhibit protein synthesis. Loading of the blot was normalized for equal intensities of BRCA1 bands at time zero. Quantification of the levels of BRCA1 protein were done using NIH ImageJ software (http://imagej.nih.gov/ij/). Result represents the mean ± SD of at least three independent experiments. *p < 0.05; **p < 0.01; ***p < 0.001. **G.** Cells were treated with 10 μM of MG132 for 6 h, and then subjected to western blot analysis with an anti-BRCA1 antibody. **H.** Cells were treated with 10 μM of MG132 for 6 h, and then subjected to immunoprecipitation with an anti-BRCA1 antibody, followed by western blot analysis with an anti-ubiquitin antibody. BRCA1 levels were normalized by loading different amounts of protein lysates prior to immunoprecipitation**. I.** Lysate from control or Rak-depleted MCF10A cells were immunoprecipitated with an anti-BRCA1 antibody or a mouse IgG and then subjected to western blot analysis. Protein levels of BRCA1 were normalized before pull-down**. J.** MCF10A cells were transfected with control siRNA, Rak siRNA, UBE2T siRNA alone or in combination. Cell lysates were immunoprecipitated with an anti-BRCA1 antibody and then subjected to western blot analysis with an anti-ubiquitin antibody. Protein levels of BRCA1 were normalized before pull-down. **K.** Representative immunohistochemical staining of Rak and BRCA1 on breast cancer tissue microarrays (upper). A graph showing a positive correlation between Rak and BRCA1 protein expression on breast cancer tissue microarrays **(***n* **= 75) (***Pearson’s r = 0.707759; p < 0.05)***.**

In order to investigate how Rak is involved in DSB repair, we examined the level of several DNA repair-related proteins involved in both the HR and NHEJ repair pathways. We found that Rak deficiency significantly reduced the level of BRCA1 protein (Figure 3A) without affecting mRNA level (Figure 3B), suggesting that Rak may regulate BRCA1 at the posttranslational level. Since expression of BRCA1 is regulated in a cell cycle-dependent fashion [51], we examined whether Rak deficiency-induced reduction of BRCA1 is due to alterations in cell cycle distribution, but found no significant difference in cell cycle distribution between control and Rak-depleted MCF10A cells (Supplementary Information, Figure S3). This suggests that reduced BRCA1 protein levels caused by Rak deficiency is not due to the cell cycle alterations.

Next, we examined whether there is a physical interaction between Rak and BRCA1 and found that endogenous Rak interacts with BRCA1 (Figure 3C). To better understand their functional interplay, we tried to identify specific binding domains for Rak and BRCA1. BRCA1 contains the N-terminal RING domain essential for BRCA1′s E3 ligase activity [52], nuclear localization signal (NLS) [53] and two BRCA1 C-terminal (BRCT) domains critical for the DNA damage response pathways [54, 55]. As shown in Figure 3D, Rak was unable to bind to the BRCT deletion mutant (Δ1527–1863), suggesting that the BRCT domain is essential to mediate its interaction with Rak. Similar to other Src kinase family members, Rak contains SH2 and SH3 domains at the N-terminus and a kinase domain at the C-terminus [24]. While the SH2 domain is known to interact with phosphorylated tyrosine residue(s) of target protein, the SH3 domain mediates the interaction with target proteins through a proline-rich region [24]. As shown in Figure 3E, BRCA1 was not able to bind to the SH3 deletion mutant, indicating that the SH3 domain of Rak is important for its interaction with BRCA1. Deletion of the SH2 domain of Rak did not affect its interaction with BRCA1 (Figure 3E).

Since Rak binds to and regulates BRCA1 at the protein level, we hypothesized that Rak may regulate BRCA1 protein stability. To test this possibility, we compared BRCA1 protein turnover between control and Rak-depleted MCF10A cells in the presence of 10 μg/ml of the protein synthesis inhibitor cycloheximide (CHX). As shown in Figure 3F, Rak deficiency was sufficient to reduce the half-life of BRCA1 protein, suggesting a critical role for Rak in BRCA1 stabilization.

### Rak deficiency enhances ubiquitin-mediated BRCA1 degradation

To further test whether Rak positively regulates BRCA1 protein stability through inhibition of the 26S proteasomal pathway, we treated cells with 10 μM of the proteasome inhibitor MG132. MG132 treatment significantly increased BRCA1 protein levels in Rak-depleted MCF10A cells (Figure 3G), indicating that Rak inhibits proteasome-mediated BRCA1 degradation. Emerging evidence suggests that the ubiquitin-proteasome systems are responsible for ubiquitin-mediated BRCA1 degradation [19, 56]. In order to examine whether Rak deficiency leads to ubiquitin-mediated proteasomal degradation of BRCA1 protein, we carried out *in vivo* ubiquitination assays and found that BRCA1 was significantly ubiquitinated in the absence of Rak (Figure 3H). In addition, MG132 treatment increased BRCA1 ubiquitination (Figure 3H). This data suggests that Rak protects the BRCA1 protein from ubiquitin-mediated proteasomal degradation.

It has previously reported that ubiquitin conjugating enzyme 2T (UBE2T) interacts with and targets BRCA1 for degradation [56]. To determine whether UBE2T is responsible for Rak deficiency-induced BRCA1 ubiquitination, we examined the interaction of BRCA1 with UBE2T in the presence or absence of Rak. As shown in Figure 3I, the association between endogenous BRCA1 and UBE2T is significantly increased in the absence of Rak. It is worth noting that Rak deficiency does not alter the expression of UBE2T (data not shown). Moreover, BRCA1 ubiquitination was significantly reduced in the absence of UBE2T (Figure 3J), suggesting that Rak stabilizes BRCA1 protein thorough inhibiting the interaction of BRCA1 with UBE2T.

### A positive correlation exists between Rak and BRCA1 expression in breast cancer tissues

Since BRCA1 is destabilized in the absence of Rak, we evaluated a correlation between Rak and BRCA1 expression on breast cancer tissue microarrays by immunohistochemistry. Although the mutation status of Rak and BRCA1 is unknown, we found that there is a positive correlation between Rak and BRCA1 expression (*Pearson’s r* = 0.707759, *p* < 0.05) (Figure 3K), suggesting a potential link between Rak and BRCA1 expression in breast cancer. It is also worth noting that Rak is under-expressed in 20% (14 out of 70 cases) of breast cancer tissues.

### Rak directly phosphorylates BRCA1

**Figure 4:**
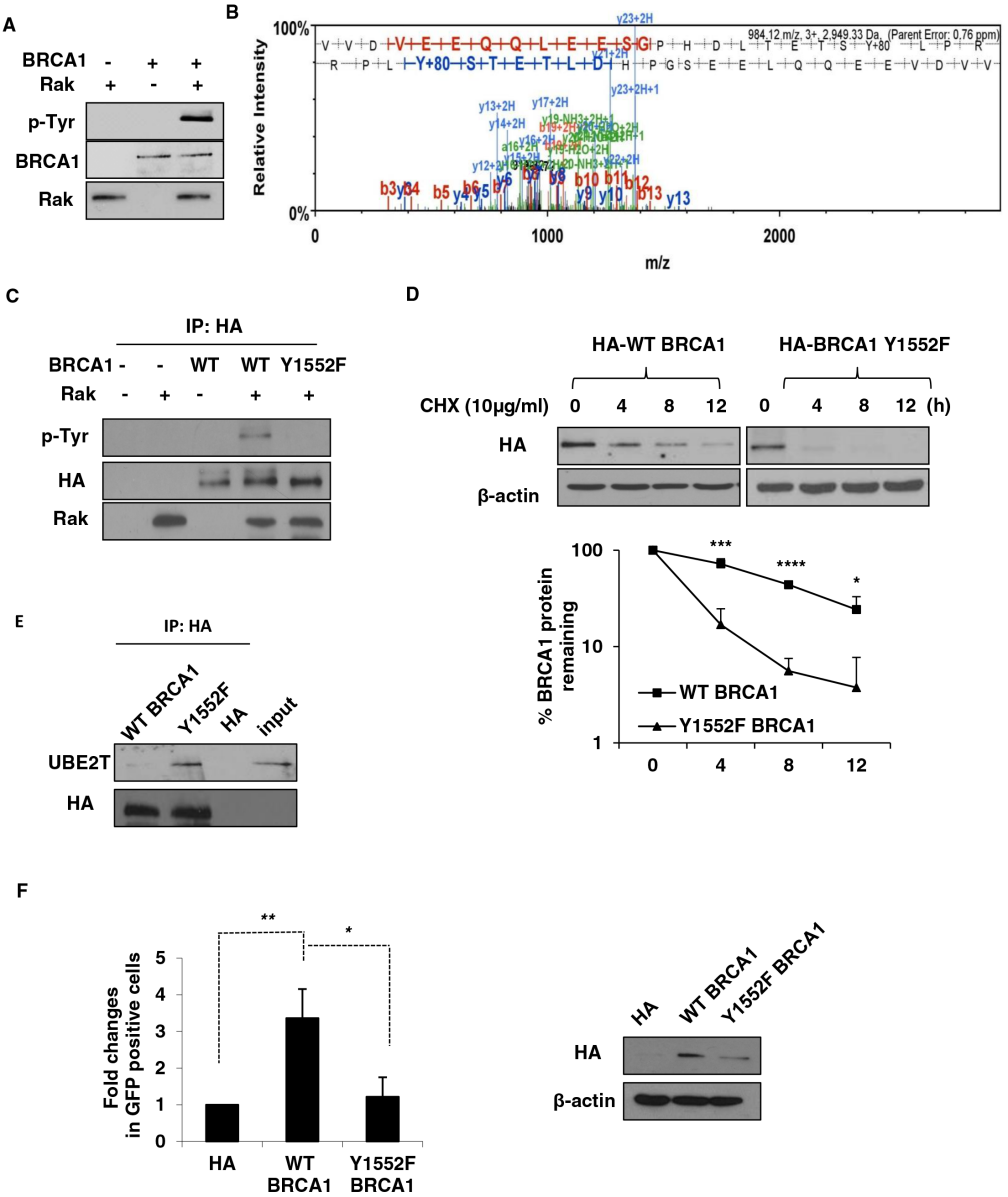
Rak-mediated tyrosine phosphorylation of BRCA1 is essential for its stability and function **A.** Purified recombinant Rak (active) was incubated with BRCA1 in kinase assay buffer, followed by western blot analysis with an anti-phospho-tyrosine antibody. **B.** Identification of BRCA1 Tyr1552 residue as a phosphorylation site by Rak. **C.** HCC1937 cells were transfected with either wild-type (WT) or Y1552F mutant BRCA1, immunoprecipitated with an anti-HA antibody and incubated with recombinant Rak in the presence of ATP. Phospho-tyrosine was determined by western blotting. Protein levels of BRCA1 were normalized before pull-down. **D.** Ectopic expression of wild-type BRCA1 or Y1552F mutant BRCA1 in HCC1937 cells was measured in the presence of CHX to inhibit protein synthesis for the indicated periods of time. Loading of the blot was normalized for equal intensities of BRCA1 bands at time zero. Quantification of the levels of BRCA1 protein were done using NIH ImageJ software (http://imagej.nih.gov/ij/). **E.** Lysates from HCC1937 cells expressing either wild-type BRCA1 or Y1552F mutant BRCA1 were immunoprecipitated with an anti-HA antibody and then subjected to western blot analysis with an anti-UBE2T antibody. Protein levels of BRCA1 were normalized before pull-down. **F.** HCC1937 cells expressing either wild-type BRCA1 or Y1552F mutant BRCA1 were subjected to HR assay. Result represents the mean ± SD of at least three independent experiments. *p < 0.05; **p < 0.01; ***p < 0.005; ****p < 0.0001.

Studies have shown that tyrosine phosphorylation-coupled ubiquitin-proteasome pathways may be a key mechanism for the regulation of protein stability [25, 57]. The interaction of Rak with BRCA1 raised the possibility that Rak might protect BRCA1 directly through tyrosine phosphorylation. To examine this possibility, we performed *in vitro* kinase assays using commercially available recombinant BRCA1 and Rak (active) proteins and found that Rak is able to phosphorylate BRCA1 (Figure 4A). Using tandem mass spectrometry, we identified one tyrosine residue, Tyr 1552, on BRCA1 that is phosphorylated by Rak (Figure 4B). In order to confirm tyrosine phosphorylation of BRCA1 on Tyr1552 by Rak, we generated a tyrosine to phenylalanine substitution mutant (Y1552F) of BRCA1 and found that the Y1552F mutant abolished Rak-mediated tyrosine phosphorylation (Figure 4C), confirming that Tyr 1552 of BRCA1 is required for phosphorylation of BRCA1 by Rak.

### Rak-mediated tyrosine phosphorylation of BRCA1 is critical for the stability and function of BRCA1

To understand the importance of Rak-mediated BRCA1 tyrosine phosphorylation on Tyr 1552 in the stability of BRCA1 protein, we expressed either wild-type or Y1552F mutant of BRCA in BRCA1-deficient HCC1937 cells and examined the half-life BRCA1. As shown in Figure 4D, BRCA1 Y1552F mutant had a significantly shorter half-life than wild-type in the presence of CHX, suggesting that Rak-mediated phosphorylation of BRCA1 on Tyr1552 is required for the stability of BRCA1. Consistent with the lack of BRCA1 phosphorylation by Rak, the interaction of Y1552F mutant of BRCA1 with UBE2T was significantly increased (Figure 4E). To determine whether this phosphorylation is critical for the function of BRCA1, we measured the HR efficiency using the pDR-GFP reporter system and found that Y1552F mutant of BRCA1 impaired HR-mediated DSB due to destabilization of BRCA1 while wild-type BRCA1 facilitated HR (Figure 4F). Collectively, our findings suggest that Rak-mediated BRCA1 tyrosine phosphorylation is important for BRCA1 protein stability and function in HR-mediated DSB repair.

### Loss of BRCA1 is partially responsible for Rak deficiency-mediated DNA damage

**Figure 5:**
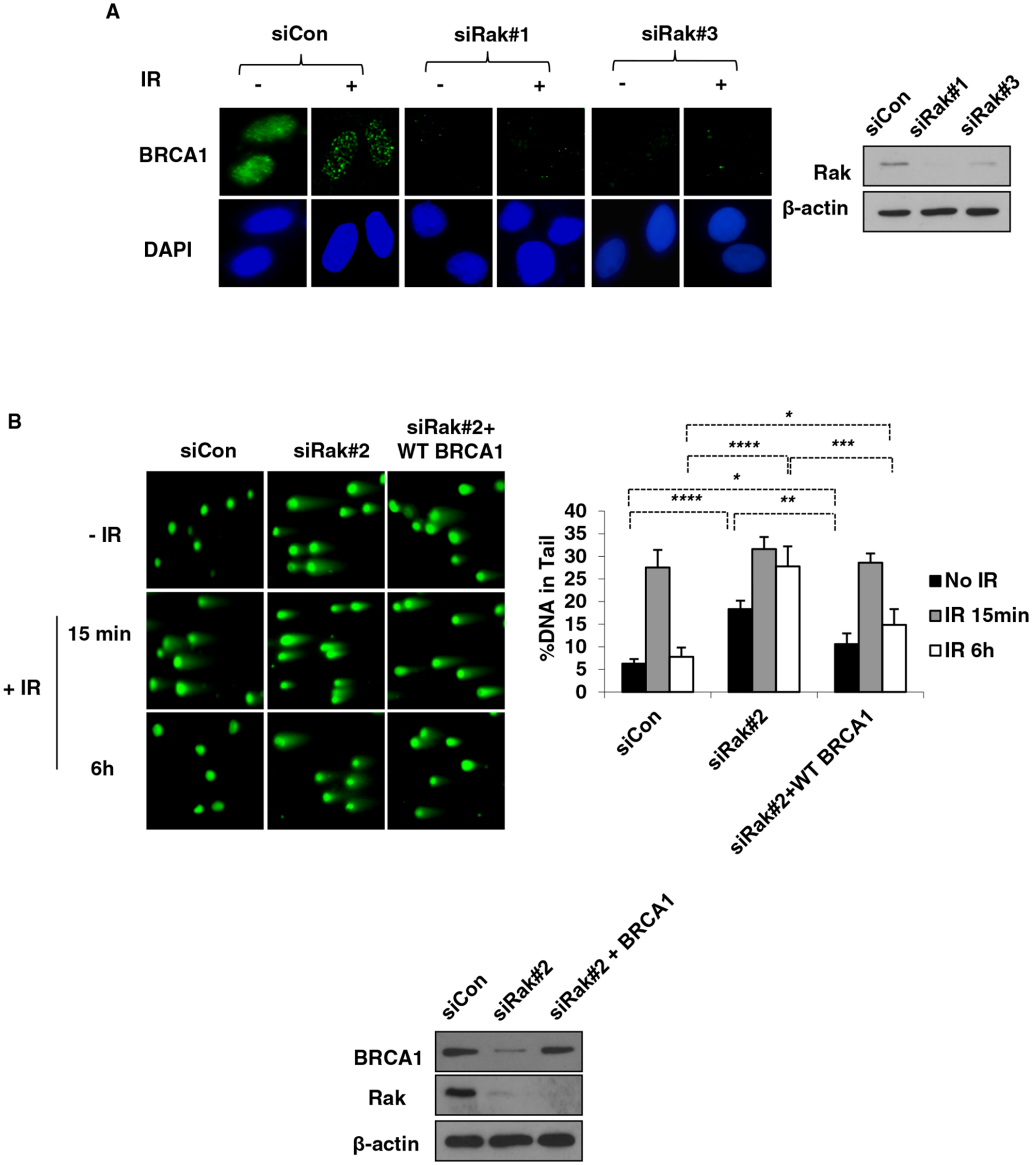

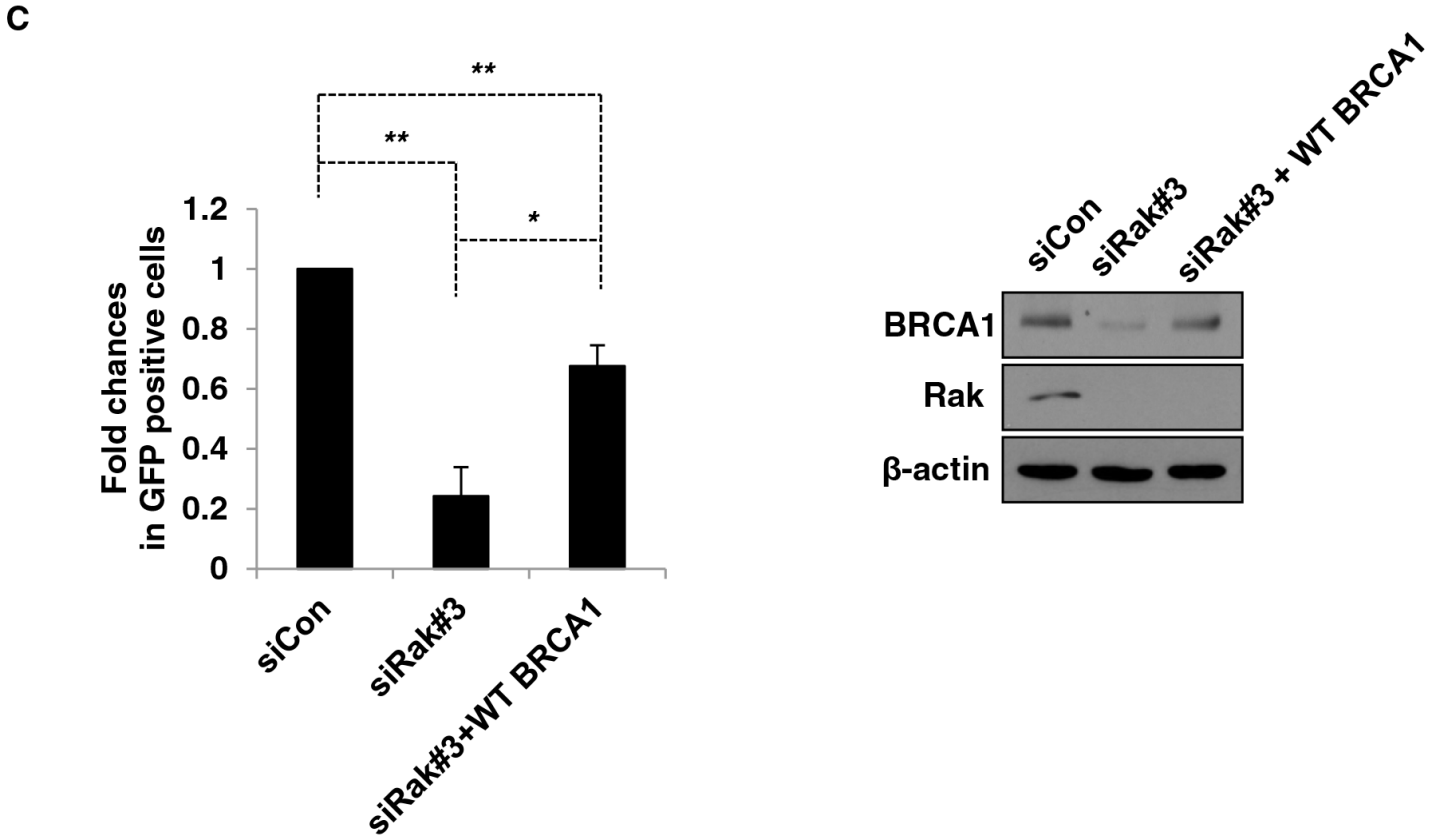
Re-expression of BRCA1 partially rescues DNA damage caused by Rak deficiency MCF10A cells were transfected with either control siRNA or Rak siRNAs. **A.** Forty eight hours after transfection, cells were irradiated with 10 Gy of IR, incubated for 1 h and stained with an anti-BRCA1 antibody, followed by incubation with Alexa 488 Fluor-conjugated secondary antibody. DAPI staining was carried out for visualizing nuclei. MCF10A cells were transfected with either control siRNA or Rak siRNA along with BRCA1 or control plasmid and subjected to **B.** the neutral pH comet assay and **C.** HR assay. Result represents the mean ± SD of at least three independent experiments. *p < 0.05; **p < 0.005; ***p < 0.0005; ****p < 0.0001

As expected, Rak deficiency-induced reduction of BRCA1 failed to recruit BRCA1 to damage sites in response to IR (Figure 5A). To determine whether loss of BRCA1 is responsible for the Rak deficiency-induced DNA damage, we re-expressed wild-type BRCA1 in Rak-depleted MCF10A cells and found that re-expression of BRCA1 partially reduced DSB accumulation (Figure 5B) and restored DSB repair function (Figure 5B and 5C) in the absence of Rak. Since Rak deficiency leads to shorter half-life of BRCA1, we were not able to see much higher of BRCA1 protein in cells although we re-expressed BRCA1. These results suggest that loss of BRCA1 may be one of the mechanisms involved in DNA damage caused by Rak deficiency and other mechanisms may also contribute to this process.

### Rak deficiency sensitizes cells to irradiation, PARP inhibitors and cisplatin

**Figure 6:**
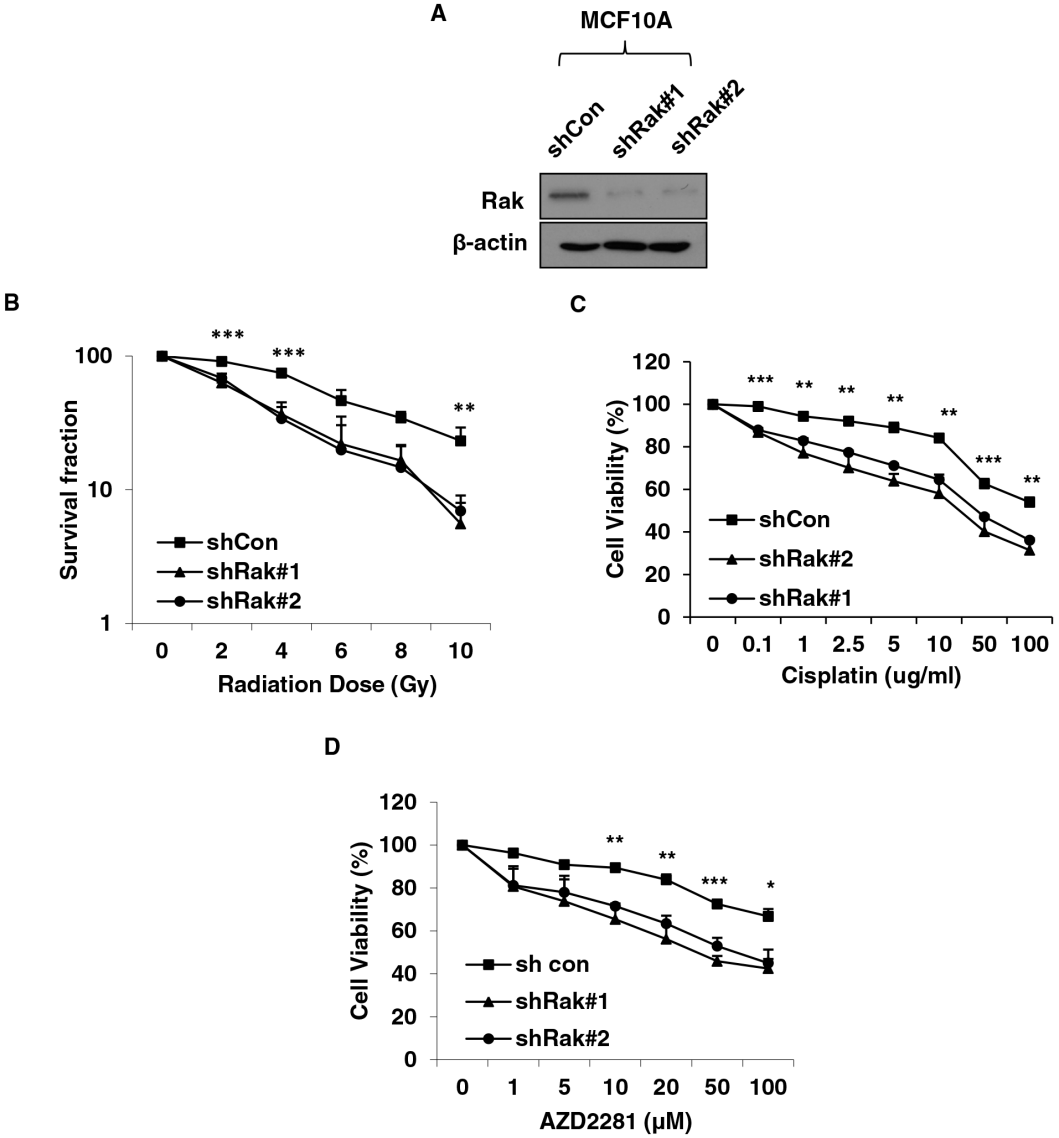
Rak deficiency confers increased vulnerability to DNA damaging agents and PARP inhibitors **A.** MCF10A cells were transfected with shRNAs targeting control or Rak and underwent puromycin selection for 3–4 weeks. Cells were subjected to western blot analysis with an anti-Rak antibody. **B.** Cells were plated at various densities in 6-well plate, irradiated at 0, 2, 4, 6, 8 or 10 Gy and subjected to clonogenic survival assays. Cells were treated with **C.** Cisplatin or **D.** PARP inhibitor AZD2281, and subjected to MTT assays. Result represents the mean ± SD of at least three independent experiments. *p < 0.05; **p < 0.01; ***p < 0.005.

Tumor cells with defective HR function have been shown to be more sensitive to DNA damaging agents and PARP inhibitors [58, 59]. Since Rak deficiency causes impairment of HR-mediated DSB repair, we hypothesized that cells lacking Rak would be more sensitive to DNA damaging agents, such as irradiation and Cisplatin, as well as PARP inhibitors. As expected, Rak-depleted MCF10A cells (Figure 6A) exhibited increased sensitivity to IR (Figure 6B), cisplatin (Figure 6C) and PARP inhibitor, AZD2281 (Olaparib) (Figure 6D). Conversely, ectopic expression of Rak in MDA-MB-231 cells, which express low levels of Rak (Supplementary Information, Figure S4A) conferred resistance to radiation (Supplementary Information, Figure S4B) and PARP inhibitor (Supplementary Information, Figure S4C). These data suggest that the level of Rak may serve as an important indicator for prediction of therapeutic effects to DNA damaging agents and PARP inhibitors.

## DISCUSSION

The protein level of BRCA1 has been often found to be low in sporadic breast cancer patients with no family history of the disease or *BRCA1* mutation [10–15], suggesting the existence of other factors or mechanisms that control the BRCA1 protein levels during mammary tumorigenesis. In this study, we, for the first time, demonstrated that Rak positively regulates the stability and function of BRCA1 via tyrosine phosphorylation of BRCA1 on Tyr1552, which in turn prevents ubiquitin-mediated BRCA1 proteasomal degradation. Accumulating evidence suggests that BRCA1 protein levels can be regulated by the ubiquitin-proteasome pathway [19, 56, 60]. The role of UBE2T has so far been described in the DNA damage-induced monoubiquitination of Fanconi anemia complementation group D2 (FANCD2) [61], which is critical for the FA pathway to function [62], and in the polyubiquitination of BRCA1 through the interaction with the BRCA1/BRCA1-associated RING domain (BARD1) heterodimer, which results in BRCA1 degradation [56]. In addition, UBE2T is overexpressed in breast cancer and silencing of UBE2T upregulates BRCA1 protein [56]. In the present study, we also found that Rak deficiency increases the interaction between UBE2T and BRCA1 and thus enhances UBE2T-mediated BRCA1 ubiquitination, although silencing of Rak does not alter the expression of UBE2T (data not shown). Since Rak directly phosphorylates BRCA1, we speculate that Rak-mediated BRCA1 tyrosine phosphorylation may inhibit the interaction of BRCA1 with UBE2T and subsequent BRCA1 degradation. We have previously demonstrated that Rak-mediated tyrosine phosphorylation of its substrate PTEN inhibits PTEN’s interaction with a HECT-type E3 ligase NEDD4–1, thereby protecting PTEN from ubiquitin-mediated proteasomal degradation [25]. Therefore, these findings highlight that Rak-mediated tyrosine phosphorylation-coupled ubiquitin-proteasome systems may play an important role in the regulation of protein stability. The NCBI ClinVar variation report reveals that duplication that leads to frameshift variation (c.4655dupA [p.Tyr1552Terfs]) (http://www.ncbi.nlm.nih.gov/clinvar/RCV000130701) and single nucleotide variant (c.4656C > G [p.Tyr1552Ter]) (http://www.ncbi.nlm.nih.gov/clinvar/RCV000048620) [63] that leads to termination were found in conditions of hereditary neoplastic syndromes and familial breast/ovarian cancer patients, respectively. Therefore, it would be interesting to examine BRCA1 Y1552 tyrosine phosphorylation on breast tumors. Monitoring *BRCA1* gene mutations has proven to be useful for breast cancer risk assessment and therapeutic decision-making. Since loss of BRCA1 protein expression has also been reported in sporadic breast cancer patients despite having intact *BRCA1* gene [13, 15], testing for an additional biomarker indicative of the presence of BRCA1, such as Rak, would provide a more precise representation of tumor characteristics and a complementary strategy, thereby improving patient outcomes.

Importantly, we found a positive correlation between Rak and BRCA1 expression in breast cancer despite not knowing information about the mutation status of Rak and BRCA1. Thus, for future studies, we will examine the expression of Rak in breast cancer cell lines and tissues with WT and mutant BRCA1 and the regulation of mutant BRCA1 stability by Rak.

Rak has been shown to play a role in cell cycle progression [24]. In this study, we found that depletion of Rak does not affect normal cell cycle progression while it impairs the G2/M checkpoint activation in response to irradiation. This suggests that different signaling pathways regulate normal cell cycle progression and DNA damage-induced checkpoint activation.

Rak deficiency results in impaired DNA damage response signaling and HR-mediated DSB repair, partly due to loss of BRCA1 and thus confers cellular hypersensitivity to DNA damaging agents, radiation and cisplatin, as well as PARP inhibitors. Therefore, it is possible that Rak deficiency could be a prognostic marker that predicts therapeutic response to radiotherapy, chemotherapy and/or PARP inhibitor therapy. As the next step, we plan to investigate the predictive value of the level of Rak in therapeutic outcomes using breast tumor tissue samples. This will allow us to determine the feasibility of using Rak as a predictive marker in a clinical setting.

**Figure 7:**
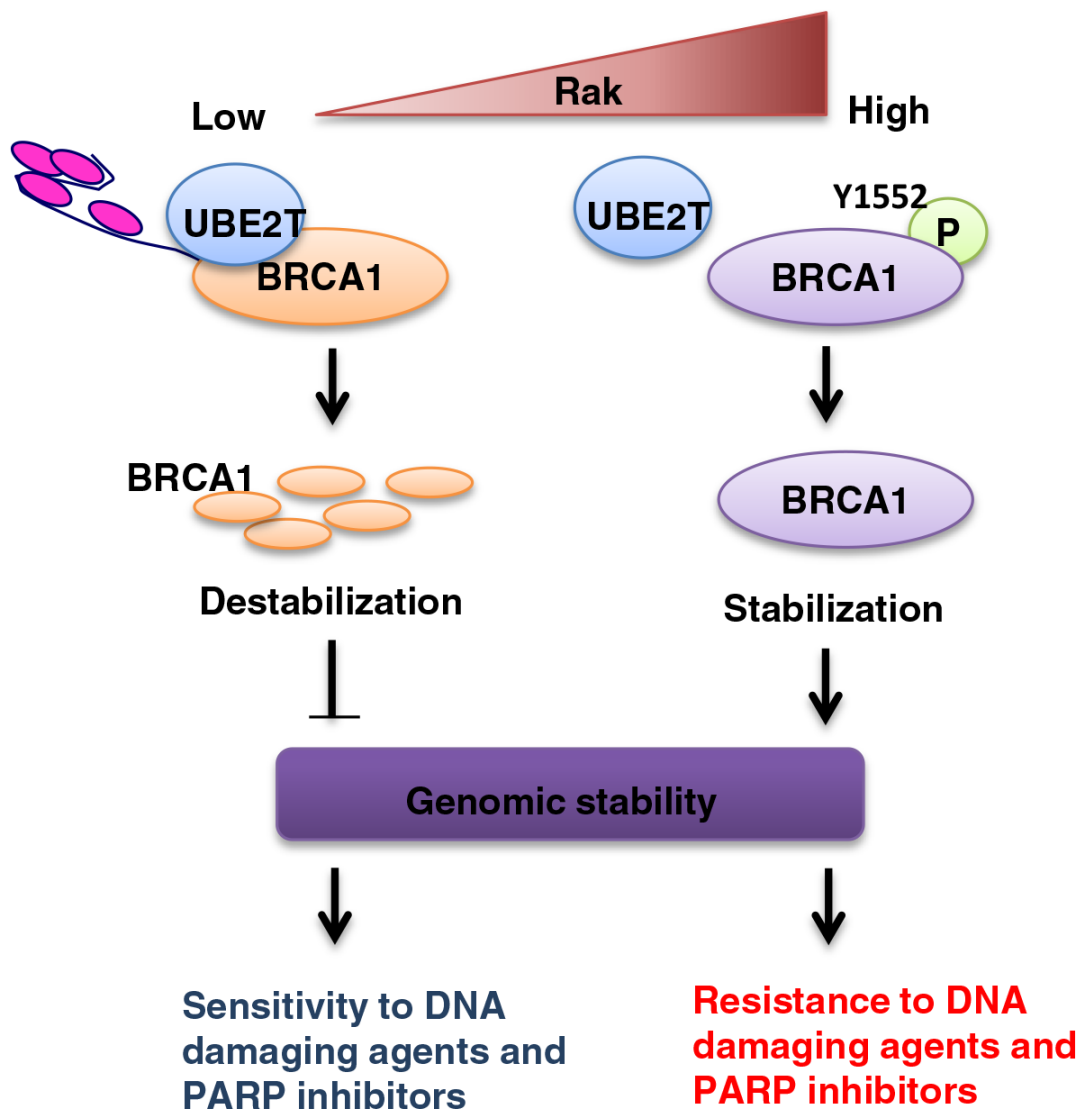
A schematic model for the role of Rak in the regulation of BRCA1 protein stability and function and possible clinical implications Rak-mediated BRCA1 tyrosine phosphorylation on Tyr1552 is critical for the stability and function of BRCA1. Rak deficiency leads to BRCA1 destabilization and subsequent genomic instability, conferring cellular sensitivity to DNA damaging agents and PARP inhibitors.

Our results, thereby, identify a critical function of Rak in the regulation of the stability and function of BRCA1 that may contribute to the maintenance of genomic stability and provide Rak as a potential prognostic indicator of therapeutic response (Figure 7).

## MATERIALS AND METHODS

### Cell culture, antibodies and chemicals

MCF10A cells were grown in Dulbecco’s Modified Eagle Medium (DMEM)/F-12 (1:1) (HyClone, Logan, UT) containing 5% horse serum, 0.5 μg/ml hydrocortisone, 20 ng/ml epidermal growth factor (EGF), 100 ng/ml insulin, 100 ng/ml cholera toxin and 1% penicillin/streptomycin solution (Themo Fisher Scientific, Waltham, MA). HEK293T, MDA-MB-231 and MDA-MB-468 cells were grown in DMEM (HyClone) containing 10% fetal bovine serum (FBS) (Geminin Bio-products, Woodland, CA) and 1% penicillin/streptomycin solution. HCC1937 cells were grown in RPMI 1640 (HyClone) containing 10% FBS and 1% penicillin/streptomycin solution. U2OS cells were grown in McCoy’s 5A medium (Hyclone) containing 10% FBS and 1% penicillin/streptomycin solution. All cell lines used in this study were obtained from the American Type Culture Collection (ATCC) (Manassas, VA) and were incubated under humidified conditions with 5% CO_2_ at 37°C.

Antibodies used in this study were: BRCA1 (EMD Millipore, Billerica, MA), Frk/Rak (Abcam, Cambridge, UK), phospho-Histone H2AX (Ser139) (EMD Millipore), phosph-Histone H3 (Ser10) (Cell Signaling Technology, Beverly, MA), phospho-tyrosine (Sigma-Aldrich, St. Louis, MO), ubiquitin (Enzo Life Sciences, Farmingdale, NY), Flag (Sigma-Aldrich), HA (Roche, Indianapolis, IN) and β-actin (Sigma-Aldrich).

Cycloheximide (CHX) and MG132 were purchased from Sigma-Aldrich and Santa Cruz Biotechnology (Santa Cruz, CA), respectively. Olaparib (AZD2281) was purchased from Selleckchem (Houston, TX).

### Western blot analysis, immunoprecipitation and immunofluorescence

Cells were harvested, washed and lysed in modified RIPA lysis buffer [50 mM Tris-HCl (pH 7.4), 150 mM NaCl, 1 mM ethylenediaminetetraacetic acid (EDTA), 1% nonyl phenoxypolyethoxylethanol (NP-40), 0.25% sodium deoxycholate, 1 mM sodium fluoride (NaF) 1 mM sodium orthovanadate, 1 mM phenylmethylsulfonyl fluoride (PMSF), 1 μg/ml aprotinin, 1 μg/ml leupeptin and 1 μg/ml pepstatin with Complete protease inhibitor cocktail (Amresco, Solon, OH) for 1 h at 4°C. Equal amounts of proteins were separated by sodium dodecyl sulfate (SDS)-polyacrylamide gel electrophoresis (PAGE) and transferred to nitrocellulose membrane. Nonspecific binding was blocked by soaking membranes in Tris-buffered saline-Tween 20 (TBS-T) buffer [50 mM Tris-HCl (pH 7.5), 150 mM NaCl and 0.1% Tween 20] containing 5% non-fat milk for 1 h. The membranes were incubated with primary antibodies, followed by incubation with secondary antibodies. Blots were developed using Pierce ECL western blotting substrate (Thermo Fisher Scientific, Waltham, MA).

Immunoprecipitation was performed by incubating lysates with 1–2 ug of antibody at 4°C overnight, followed by incubation with protein A/G-conjugated agarose beads (Santa Cruz Biotechnology). Beads were washed 4 times with ice-cold RIPA buffer, resuspended in 4x SDS-PAGE sample buffer and subjected to western blot analysis.

Cells grown on coverslips were washed with phosphate buffered saline (PBS), fixed with 4% paraformaldehyde and permeabilized in PBS containing 1% Triton X-100 and 0.5% NP-40. For blocking nonspecific binding, cells were incubated with 3% bovine serum albumin (BSA) and 1% normal horse serum in PBS-T buffer. Cells were then incubated with primary antibodies, followed by incubation with Alexa Fluor 488-conjugated secondary antibodies (Life Technologies, Grand Island, NY). Slides were mounted in Fluoroshield mounting medium containing 4′,6′-diamidino-2-phenylindole (DAPI) (Abcam) and images were taken with a confocal microscope.

### Plasmids, shRNAs, siRNAs and transfection

The Flag-tagged wild-type and deletion mutants of Rak [24] were provided by Dr. Rolf J. Craven (University of Kentucky). The HA-tagged wild-type and deletion mutants of BRCA1 [64] were a kind gift from Dr. Jeffrey Parvin (Ohio State University). pDR-GFP (plasmid 26475) and pCBASce (plasmid 26477) were purchased from Addgene (Cambridge, MA) [43, 65]. Tyrosine (Y) to phenylalanine (F) mutation in BRCA1 (BRCA1 Y1552F) was generated using the QuikChange II XL site-directed mutagenesis kit (Agilent Technologies, Santa Clara, CA), according to the manufacturer’s protocol. MISSION siRNAs for FRK/Rak and negative control were purchased from Sigma-Aldrich. Cells were transfected with siRNAs by using Oligofectamine (Invitrogen), according to the manufacturer’s protocol. Rak-specific and scrambled control shRNAs were purchased from Santa Cruz Biotechnology (Santa Cruz, CA). Cells were transfected with plasmids by using TurboFect transfection reagent (Themo Scientific), according to the manufacturer’s protocol.

### Quantitative real time-polymerase chain reaction (qRT-PCR)

Total RNAs from cells were isolated using the RNeasy® mini kit (Qiagen, Germantown, MD), and then subjected to cDNA synthesis using the RevertAid™ First Strand cDNA Synthesis Kit (Fermentas Life Sciences Europe, Bremen, Germany), according to the manufacturer’s protocol. PCR amplification was carried out at 95°C for 5 min and 30 sec followed by 40 cycles of 15 sec at 95°C and 1 min at 60°C using the iCycler iQ5 real-time PCR detection system (Bio-Rad, Hercules, CA) and an iQ™ SYBR^R^ Green supermix (Bio-Rad). The sequences of the primer pairs were as follows: BRCA1 5′-cataggacaatggcttccatg-3′ and 5′-ctacactgtccaacacccactctc-3′ [66]; *β-actin* 5′-ctacgtcgccctggacttcgagc-3′ and 5′-gatggagccgccgatccacacgg-3′ [67]. Data analysis was carried out using the ΔΔ Ct method [68].

### Irradiation

Cells were irradiated using the RS-2000 Biological X-ray Irradiator (Rad Source Technologies, Suwanee, GA) operated at 160kV and 25mA with a dose of 1.9 Gy/min.

### Comet assay

Cells were treated with 10 Gy of IR, harvested 15 min or 6 h post-IR exposure and subjected to the neutral comet assay using Trevigen’s Comet Assay kit (Trevigen, Gaithersburg, MD), according to the manufacturer’s instruction. The images were analyzed using the CometScore software (TriTek Corp, Sumerduck, VA) and the percentage of DNA in tail (% tail DNA) was used to evaluate each comet.

### HR and NHEJ repair analysis

HR repair efficiency was determined using a pDR-GFP reporter system with [46, 47] or without modifications [69, 70]. HR-repaired GFP^+^ cells were analyzed using FACSCantoII (BD biosciences, San Jose, CA) with FlowJo software (Tree Star Inc, Ashland, OR) at the Loyola University Chicago FACS facility. PCR-based NHEJ/digest assay was carried out as described previously [50]. The following primer was used for the PCR: DR-GFP (5′-ctgctaaccatgttcatgcc-3′ and 5′-aagtcgtgctgcttcatgtg-3′).

### G2/M checkpoint assay

The G2/M cell cycle checkpoint assay was performed as described previously [44, 71]. Briefly, one hour after exposure to 3 Gy of IR, cells were fixed and incubated with an anti-phospho-histone H3 (Ser10) antibody for 1 h at room temperature, followed by incubation with Alexa Fluor 488-conjugated secondary antibody. Cells were then incubated in PBS containing 10 μg/ml RNase A and 20 μg/ml propidium iodide (PI) for 30 min. Analysis was done using FACSCantoII with FlowJo software (Tree Star Inc, Ashland, OR) at the Loyola University Chicago FACS facility.

### Tissue microarrays and immunohistochemistry

Breast cancer tissue microarrays (BR1504) were purchased from US Biomax (Rockville, MD). Clinicopathological information for the breast cancer tissue microarray is available from US Biomax (http://www.biomax.us/tissue-arrays/Breast/BR1504). For immunostaining, slides were deparaffinized and rehydrated using a series of ethanol and subjected to heat-induced antigen retrieval [72]. Slides were then incubated with either an anti-BRCA1 or an anti-Rak antibody for 1 h at room temperature, followed by incubation with secondary biotinylated antibody and the Avidin Biotin complex (ABC) (Vector Laboratories, Burlingame, CA), according to the manufacturer’s instructions. After developing color with diaminobenzidine (DAB), the slides were independently blind-reviewed by 3 authors and the intensity of staining was assessed on a scale of 0–3+ (0, no staining; 1+, weak staining; 2+, moderate staining; and 3+, strong staining).

### Recombinant proteins and *in vitro* kinase assay

Recombinant Rak (active) and BRCA1 proteins were purchased from Abcam. Kinase reactions were performed by incubating recombinant BRCA with or without recombinant Rak in kinase assay buffer containing 10 mM Tris-HCl (pH 7.4), 10 mM MgCl_2_, 1.2 mM MnCl_2_, 2 mM sodium orthovanadate, and 10 uM ATP for 30 min at 30°C and were terminated by adding SDS-PAGE sample buffer and boiling at 100°C for 5 min. The mixtures were subjected to western blot analysis.

### Tandem mass spectrometry

In order to identify potential tyrosine phosphorylation sites on BRCA1 by Rak, tandem mass spectrometry was carried out at the University of Arkansas Proteomics Core facility.

### Cell viability assay

Cellular proliferation and toxicity were determined by using a colorimetric assay based on the uptake of 3-(4,5-Dimethylthiazol-yl)-diphenyl tetrazoliumbromide (MTT) in cells. After exposure to PARP inhibitors, cells were incubated with 40 μl of MTT solution (2 mg/ml) for 4 h at 37°C. The formazan crystals were dissolved in 100 μl of DMSO and its absorbance was measured at 595 nm.

### Clonogenic survival assay

About 2–3 weeks after exposure to IR with various doses (0, 2, 4, 6, 8 or 10 Gy), colonies were fixed with methanol, stained with 0.05% crystal violet and counted.

### Statistical analysis

The results were expressed as means ± SD for each group in at least three independent experiments. Student’s *t*-test was used for two group comparisons with equal variance assumptions. Statistical analyses were performed using SAS software package (SAS Institute Inc, Cary, NC). *p*-value < 0.05 was considered statistically significant.

## SUPPLEMENTARY MATERIALS FIGURES


