# Supplementary material for: The role of Rak in the regulation of stability and function of BRCA1
Source: Oncotarget. 2015 Oct 14;8(49):86799–815. doi: 10.18632/oncotarget.5717 (PMC5689726; doi:10.18632/oncotarget.5717)
Supplement: Supplementary file 1 [file oncotarget-08-86799-s001.pdf]

SUPPLEMENTARY FIGURES

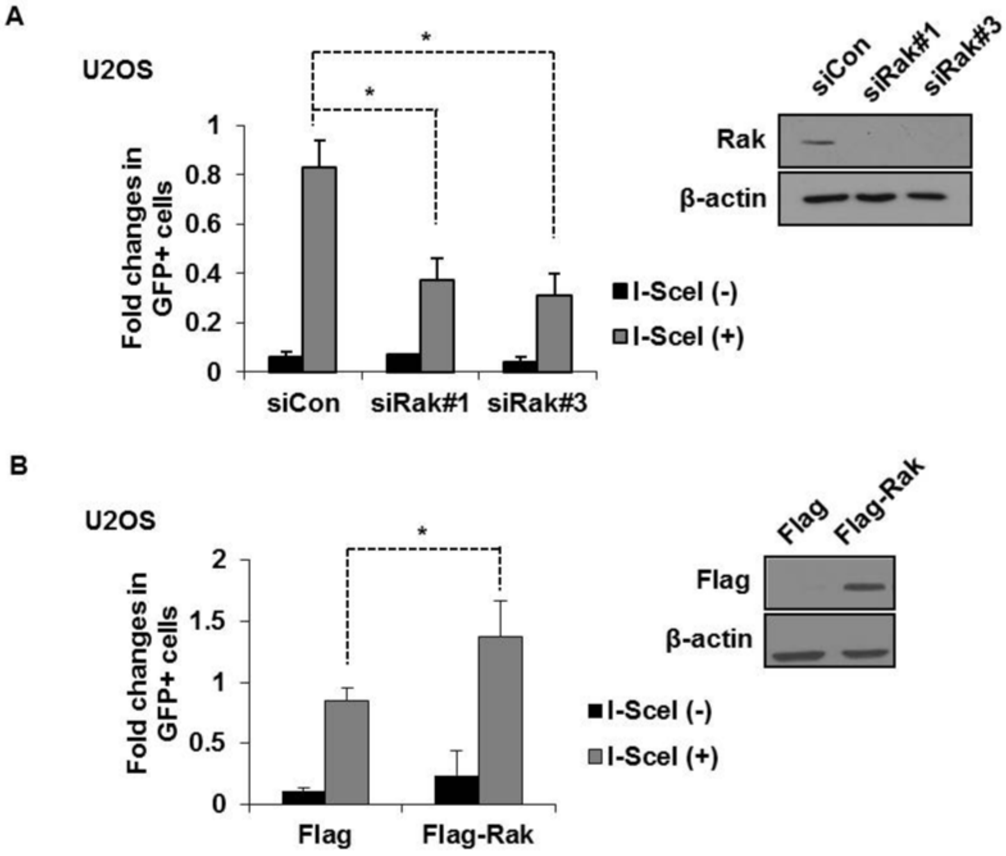

Supplementary Figure S1: Figure S1, related to Figure 2. Rak plays an important role in HR-mediated DSB repair. Twenty four hours after transfection of **A.** control siRNA or Rak siRNAs or **B.** Flag or Flag-Rak into U2OS cells stably expressing pDR-GFP, either pCBASce (I-*Sce*I expressing vector) or pCACGS (empty vector) was transfected into cells to produce a DSB within the Sce-GFP. Cells were harvested to analyze HR-repaired GFP-positive cells by FACS. Result represents the mean ± SD of at least three independent experiments. *$p < 0.05$

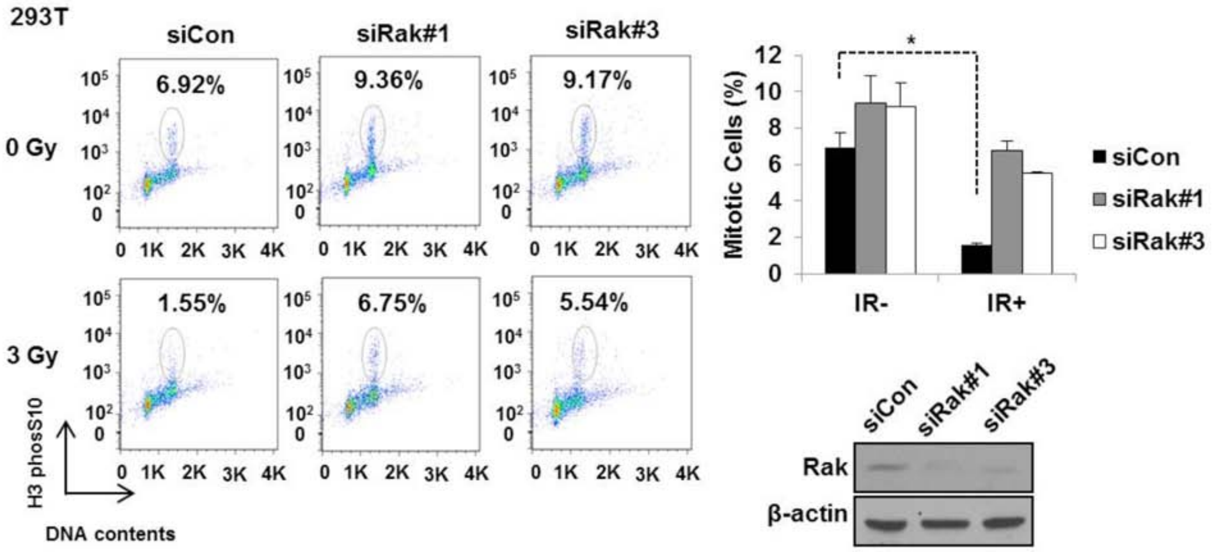

Supplementary Figure S2: Figure S2, related to Figure 2. Rak deficiency impairs G2/M cell cycle checkpoint. 293T cells were irradiated with 3 Gy of IR and then incubated for 1 h. Mitotic cells were determined by staining with an anti-phospho-histone H3 antibody followed by incubation with FITC-conjugated secondary antibody and propidium iodide. The percentage of cells in M-phase was analyzed by FACS. Result represents the mean ± SD of at least three independent experiments. *$p < 0.0005$

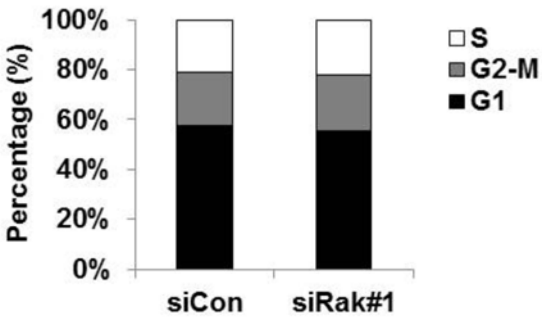

Supplementary Figure S3: Figure S3, related to Figure 3. Rak deficiency does not significantly alter the cell cycle progression. MCF10A cells were transfected with either control siRNA or Rak siRNA. After 48 h of transfection, cells were subjected to FACS analysis.

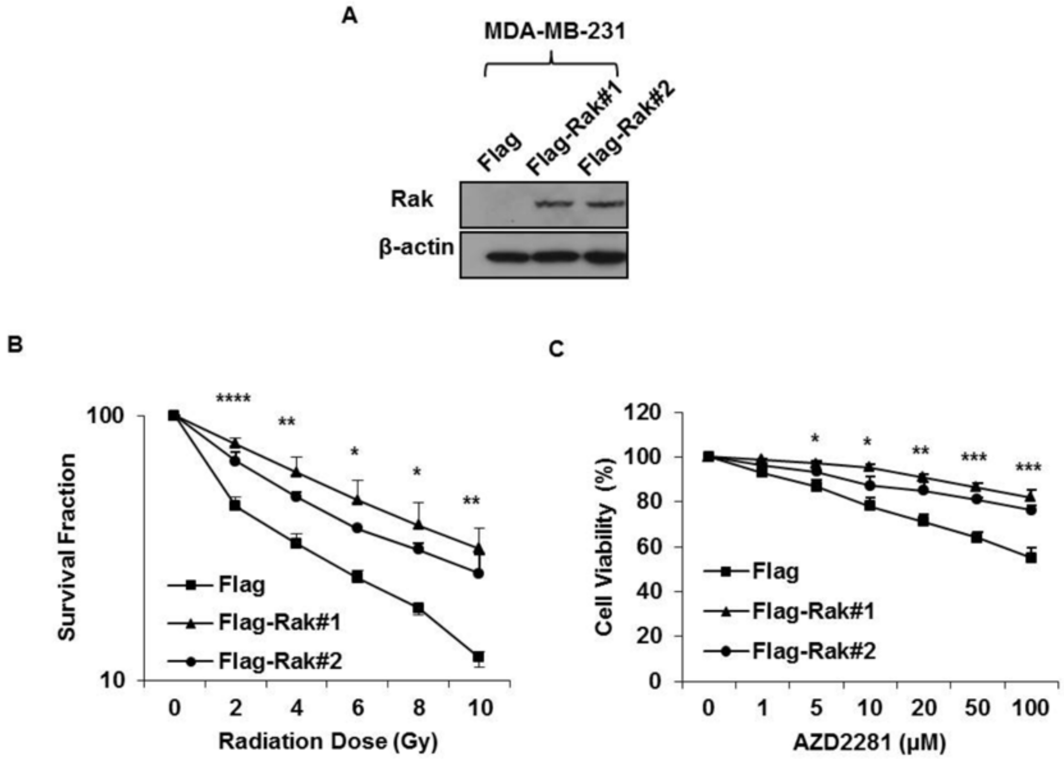

Supplementary Figure S4: Figure S4, related to Figure 3. High levels of Rak confer resistance to DNA damaging agent and PARP inhibitor. A. MDA-MB-231 cells were transfected with Flag or Flag-Rak and underwent G418 selection for 2–3 weeks. Cells were subjected to western blot analysis with an anti-Rak antibody. β-actin was used as a loading control. **B.** Cells were plated at various densities in 6-well plate, irradiated at 0, 2, 4, 6, 8 or 10 Gy and subjected to clonogenic survival assays. **C.** Cells were treated with AZD2281 and then subjected to MTT assays. Result represents the mean ± SD of at least three independent experiments. $^{*}p < 0.05$; $^{**}p < 0.005$; $^{***}p < 0.0005$; $^{****}p < 0.00001$
